# Magnetic Particles with Polymeric Shells Bearing Cholesterol Moieties Sensitize Breast Cancer Cells to Low Doses of Doxorubicin

**DOI:** 10.3390/ijms22094898

**Published:** 2021-05-05

**Authors:** Karolina H. Markiewicz, Katarzyna Niemirowicz-Laskowska, Dawid Szymczuk, Kacper Makarewicz, Iwona Misztalewska-Turkowicz, Przemysław Wielgat, Anna M. Majcher-Fitas, Sylwia Milewska, Halina Car, Agnieszka Z. Wilczewska

**Affiliations:** 1 Faculty of Chemistry, University of Bialystok, Ciolkowskiego 1k, 15-245 Bialystok, Poland; d.szymczuk@uwb.edu.pl (D.S.); 95kacper95@gmail.com (K.M.); i.misztalewska@uwb.edu.pl (I.M.-T.); 2 Department of Experimental Pharmacology, Medical University of Bialystok, Szpitalna 37, 15-361 Bialystok, Poland; sylwia.milewska@umb.edu.pl (S.M.); halina.car@umb.edu.pl (H.C.); 3 Doctoral School of Exact and Natural Sciences, University of Bialystok, 15-245 Bialystok, Poland; 4 Department of Clinical Pharmacology, Medical University of Bialystok, Waszyngtona 15A, 15-274 Bialystok, Poland; przemyslaw.wielgat@umb.edu.pl; 5 Faculty of Physics, Astronomy and Applied Computer Science, Jagiellonian University, Łojasiewicza 11, 30-348 Krakow, Poland; anna.majcher@uj.edu.pl

**Keywords:** polymer–iron oxide particles, doxorubicin, anticancer activity, combination therapy

## Abstract

One of the promising strategies for improvement of cancer treatment is application of a combination therapy. The aim of this study was to investigate the anticancer activity of nanoformulations containing doxorubicin and iron oxide particles covered with polymeric shells bearing cholesterol moieties. It was postulated that due to high affinity to cell membranes, particles comprising poly(cholesteryl acrylate) can sensitize cancer cells to doxorubicin chemotherapy. The performed analyses revealed that the developed systems are effective against the human breast cancer cell lines MCF-7 and MDA-MB-231 even at low doses of the active compound applied (0.5 µM). Additionally, high compatibility and lack of toxicity of the tested materials against human red blood cells, immune (monocytic THP-1) cells, and cardiomyocyte H9C2(2-1) cells was demonstrated. Synergistic effects observed upon administration of doxorubicin with polymer–iron oxide hybrids comprising poly(cholesteryl acrylate) may provide an opportunity to limit toxicity of the drug and to improve its therapeutic efficiency at the same time.

## 1. Introduction

Cancer is one of the leading causes of death worldwide [1]. Currently, different ways of malignancy treatment, including chemotherapy, immunotherapy, radiotherapy, and surgery, might be employed. However, despite the progress in cancer treatment made during the past few decades, resistance to conventional chemotherapeutic agents remains a major problem [2,3]. It is established that tumor growth and development is associated with combined effects of diverse factors. Therefore, methods dedicated to treatment of cancers which are based on a single ‘magic bullet’ are suboptimal. The application of a combination therapy might exert synergistic or additive effect and improve anticancer efficacy in comparison with a single chemical drug-based chemotherapy. In effect, searching for new strategies of combination therapy, such as sensitization of cancer cells to chemotherapeutic drugs by using small molecules, nanoparticles or physical techniques (radiation, phototherapy), is of great importance [4,5]. The concept of sensitization engages reversing the process of insensitivity or, if it is not effective, inhibiting an alternative pathway to facilitate the death of cancer cells using either the same drug or different drugs. Importantly, rational combinatory treatment leads to drug dose reduction, which is beneficial for patients as it limits risk of side effects [6]. It should also be emphasized that application of combination treatment, especially the sensitization strategy, is strongly justified due to the fact that targets to develop new drugs are running out [7].

Combined therapies present an opportunity to use nanoparticle-based systems. The structural features of nanoparticles make them an excellent mode for targeting and penetrating cancer cells [8]. Among various nanoparticles, iron oxides are the most frequently studied in the field of cancer diagnosis (magnetic resonance imaging, MRI) and therapy (hyperthermia, drug delivery). It is primarily due to their magnetic properties, safety, high targetability, and relatively easy synthesis and surface functionalization [9]. Our previous studies revealed that nanosystems based on amino silica-modified iron oxide nanoparticles significantly improve the killing efficiency of ceragenin, decreasing the viability of lung and colon cancer cells [10]. Recently, we also synthesized thermoresponsive cholesterol end-capped poly(*N*-isopropylacrylamide)s and evaluated their cytotoxic effect on glioblastoma cells [11]. The studies showed that the cholesteryl moiety present at the end of the PNIPAAm chain acts as a cell-penetrating agent which enables disruption of the plasma membrane and in effect leads to the restriction of tumor growth. These results suggest that polymers comprising cholesterol derivatives can be developed not only as drug carriers but also as components of combination therapy.

In this regard, in agreement with the benefits of combination therapy, we hypothesized that iron oxide nanoparticles functionalized with polymeric shell-comprising cholesterol moieties can sensitize cancer cells to chemotherapy with doxorubicin applied in low and non-toxic doses. Doxorubicin (DOX) is one of the most widely used antineoplastic agents [12]. However, it causes serious injuries of non-targeted tissues, including heart, kidneys, and brain [13,14]. Therefore, doxorubicin dose limitation is strongly advocated to reduce adverse effects of the drug, especially its cardiotoxicity [15,16].

Herein, the formation and characterization of polymer–iron oxide hybrids comprising poly(*N*-isopropylacrylamide) or poly(*N*-vinylcaprolactam) block and poly(cholesteryl acrylate) block is presented. The efficiency of nanoformulations combined by simple mixing of low doses of DOX with the obtained hybrids against human breast cancer cell lines MCF-7 and MDA-MB-231 was demonstrated. Moreover, lack of toxicity of these formulations against representative host cells (human red blood cells), immune cells (monocytic THP-1 cells), and cardiomyocyte cells (H9C2(2-1)) was confirmed.

## 2. Results

### 2.1. Formation and Characterization of Polymer-Coated Iron Oxide Particles

Polymer-coated magnetic particles were synthesized by surface-initiated RAFT (reversible addition–fragmentation chain transfer) polymerizations from dithiocarbonate-functionalized iron oxide particles (MNP@X). MNP@X particles were obtained according to the previously reported procedure [17,18]. Briefly, the four-step synthetic route involved: (1) coprecipitation of iron oxide nanoparticles from a Fe(II) and Fe(III) salts solution in the presence of ammonium base; (2) amino silica shell formation; (3) reaction of terminal amine groups with 2-bromopropionyl bromide; and (4) nucleophilic substitution with potassium *O*-ethyl carbonodithioate. Physicochemical characterization of the bare and as functionalized particles (FT IR, TEM/EDX, XRD, magnetization measurements) was reported previously [17,18,19]. Superparamagnetic iron oxide cores (magnetite and/or maghemite) of an average diameter of 10 ± 2 nm were obtained in the first step. The next stages led to the formation of aggregates of iron oxide cores (~100 nm) surrounded by a xanthate-terminated silica shell.

**Scheme 1:**
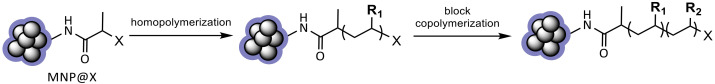
Formation of polymer–magnetic hybrids.

**Figure 1:**
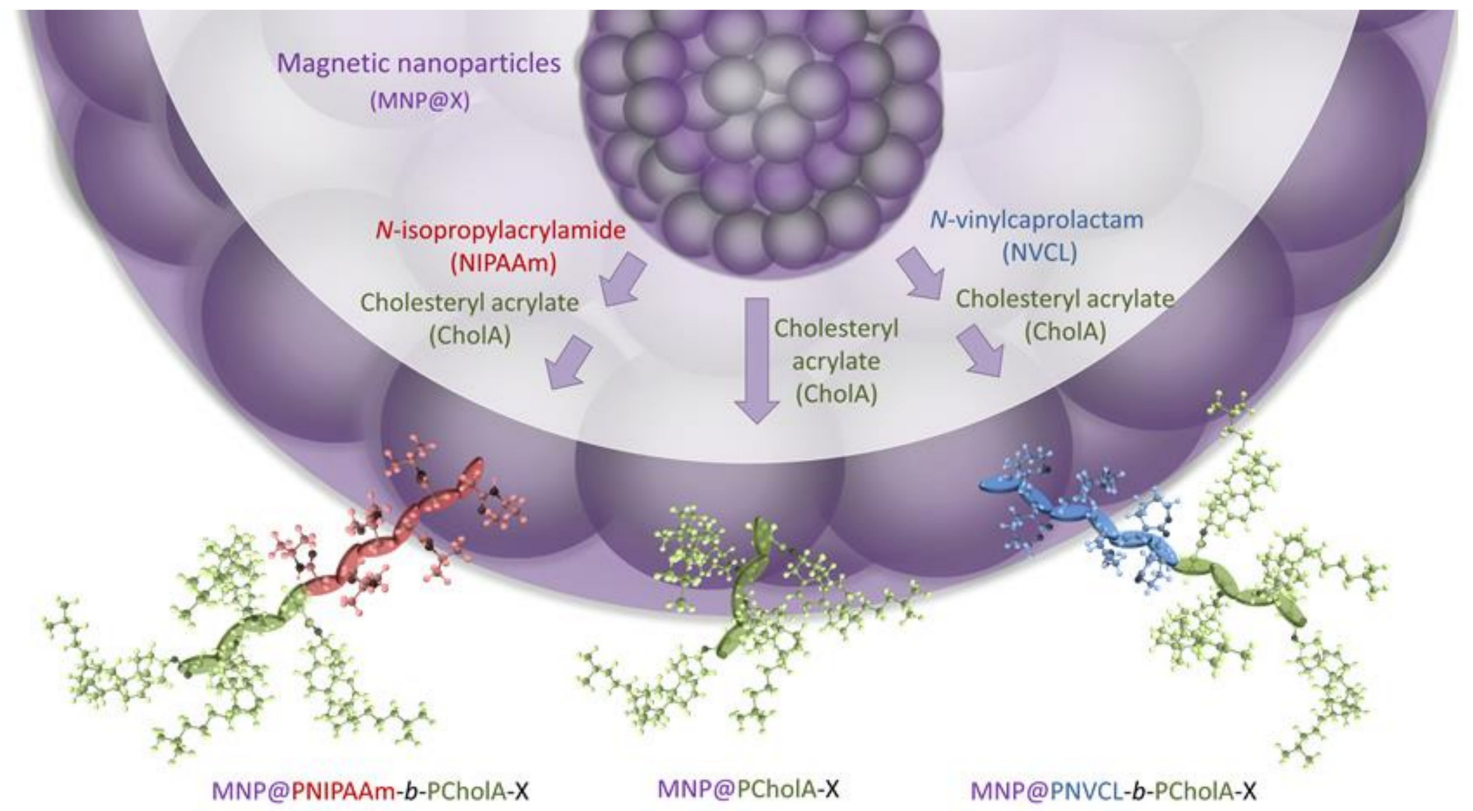
Types of polymeric shells formed on the surface of MNP@X.

MNP@X particles were used as chain transfer agents in polymerizations of *N*-isopropylacrylamide (NIPAAm) or *N*-vinylcaprolactam (NVCL) to obtain polymer–magnetic hybrids (Scheme 1). Then, block co-polymerizations with synthesized cholesteryl acrylate (CholA) (Figure S1) were performed to obtain cholesterol-modified polymer–iron oxide particles. The types of polymeric shells formed on the surface of MNP@X are presented in Table 1 and Figure 1.

**Figure 2:**
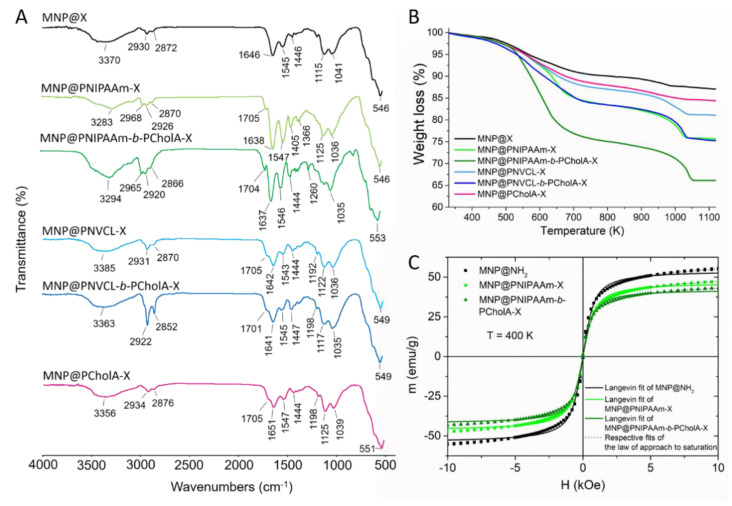
(**A**) FT IR spectra and (**B**) TG curves of the modified iron oxide particles. (**C**) Magnetization of chosen samples at 400 K with fits of Langevin function and law of approach to saturation.

The ATR FT IR spectra of the obtained nanohybrids are presented in Figure 2A. In general, one can see changes in the IR spectra of all the polymer-modified particles in comparison to the IR spectrum of the starting material (MNP@X). In the IR spectra of the products containing poly(*N*-isopropylacrylamide) or poly(*N*-vinylcaprolactam), the increase of the intensity of the bands related to the C–H aliphatic stretch (3000–2850 cm^−1^), the C=O stretch (amide I, 1640 cm^−1^), and the C–N stretch (amide II, 1540 cm^−1^) can be observed. In the spectra of the nanohybrids comprising poly(cholesteryl acrylate), the intensity of the signals characteristic of the C–H stretching vibrations (3000–2850 cm^−1^) increased.

The TG curves of the obtained hybrids are presented in Figure 2B. Iron oxide cores do not decompose in the applied temperature range, therefore, the observed weight loss is due to decomposition of the coating. The TG curve of the starting material (MNP@X) shows the total weight loss equal to 13%, which is related to the degradation of dithiocarbonate-modified amino silica shell. After polymerizations, the total weight loss in all the samples is higher (Table 1), confirming presence of polymers in the studied materials. In general, all polymer-modified MNP decompose in two broad temperature ranges (470–670 and 920–1070 K), which are related to PNIPAAm, PNVCL, and PCholA depolymerization processes.

Magnetic properties of the chosen nanoparticles (MNP@NH_2_, MNP@PNIPAAm-X, and MNP@PNIPAAm-*b*-PcholA-X) were studied in the temperature range 5–400 K and in magnetic fields up to 10 kOe. It was necessary to increase the temperature for all the analyzed samples in order to observe unequivocal superparamagnetic behavior (closing of hysteretic loops and zfc/fc magnetization convergence). The blocking temperature did not change significantly between the samples and remained close to 400 K in all the cases (Figure S2). However, closing of the hysteretic loops is visible above 300 K for all the samples (Figure S3). A single Langevin function was fitted to the data obtained at 400 K to roughly estimate the size of the nanoparticles (Figure 2C, Table S1) [20,21]. The size of the iron oxide cores was the same for all the samples (~17–18 nm) within the margin of error (Table S1). The law of approach to saturation (LA) [22,23] was used to determine the values of saturation magnetization at both 300 and 400 K; fits were performed within the range 5–10 kOe (Figure S4, Figure 2C, Table S1). As expected, saturation magnetization decreased with decreasing magnetic core mass contribution.

**Figure 3:**
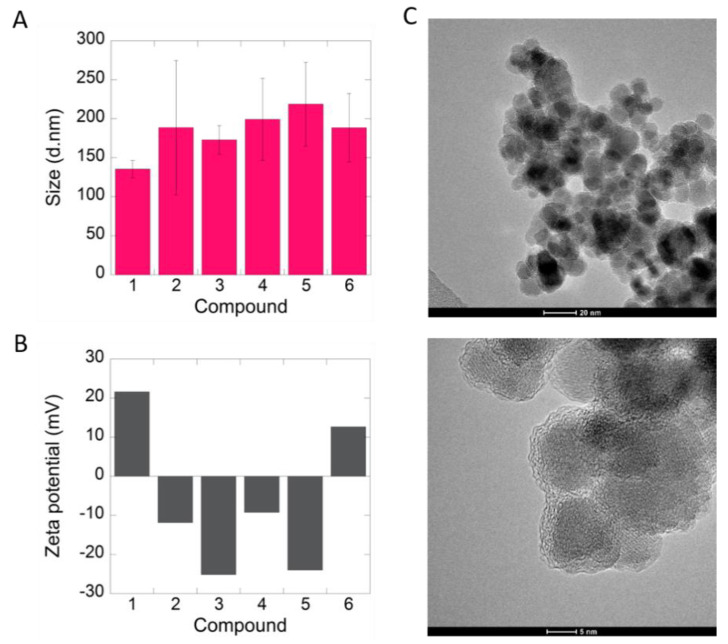
(**A**) DLS and (**B**) zeta potential results of modified iron oxide particles: 1—MNP@NH_2_, 2—MNP@PNIPAAm-X, 3—MNP@PNIPAAm-*b*-PCholA-X, 4—MNP@PNVCL-X, 5—MNP@PNVCL-*b*-PCholA-X, 6—MNP@PCholA-X. (**C**) TEM images of MNP@PNIPAAm-*b*-PCholA-X particles.

The modified particles were investigated by DLS (Figure 3A, Figure S5). DLS measurements taken in water at 25 °C revealed formation of objects of sizes ranging from 130 to 220 nm. In general, the higher the content of polymer fraction in the material, the higher the hydrodynamic diameter of the particles observed. Additionally, ζ-potential was measured to determine stability of the systems in water (Figure 3B). For the MNP@NH_2_ sample, the observed zeta potential value was equal to 21 mV. For the samples containing homopolymeric shells, MNP@PNIPAAm-X, MNP@PNVCL-X, and MNP@PCholA-X, the values decreased to −12, −9, and 12 mV, respectively. Formation of the second poly(cholesteryl acrylate) block led to further reduction in the zeta potential values to −25 for MNP@PNIPAAm-*b*-PCholA-X and −24 mV for the MNP@PNVCL-*b*-PCholA-X sample.

Representative TEM images of MNP@PNIPAAm-*b*-PCholA-X particles with two different magnitudes are presented in Figure 3C. It can be seen that iron oxide particles of spherical shape and sizes around 10–15 nm form larger aggregates (100–200 nm) covered by a thin polymer layer.

### 2.2. Biological Activity

**Figure 4:**
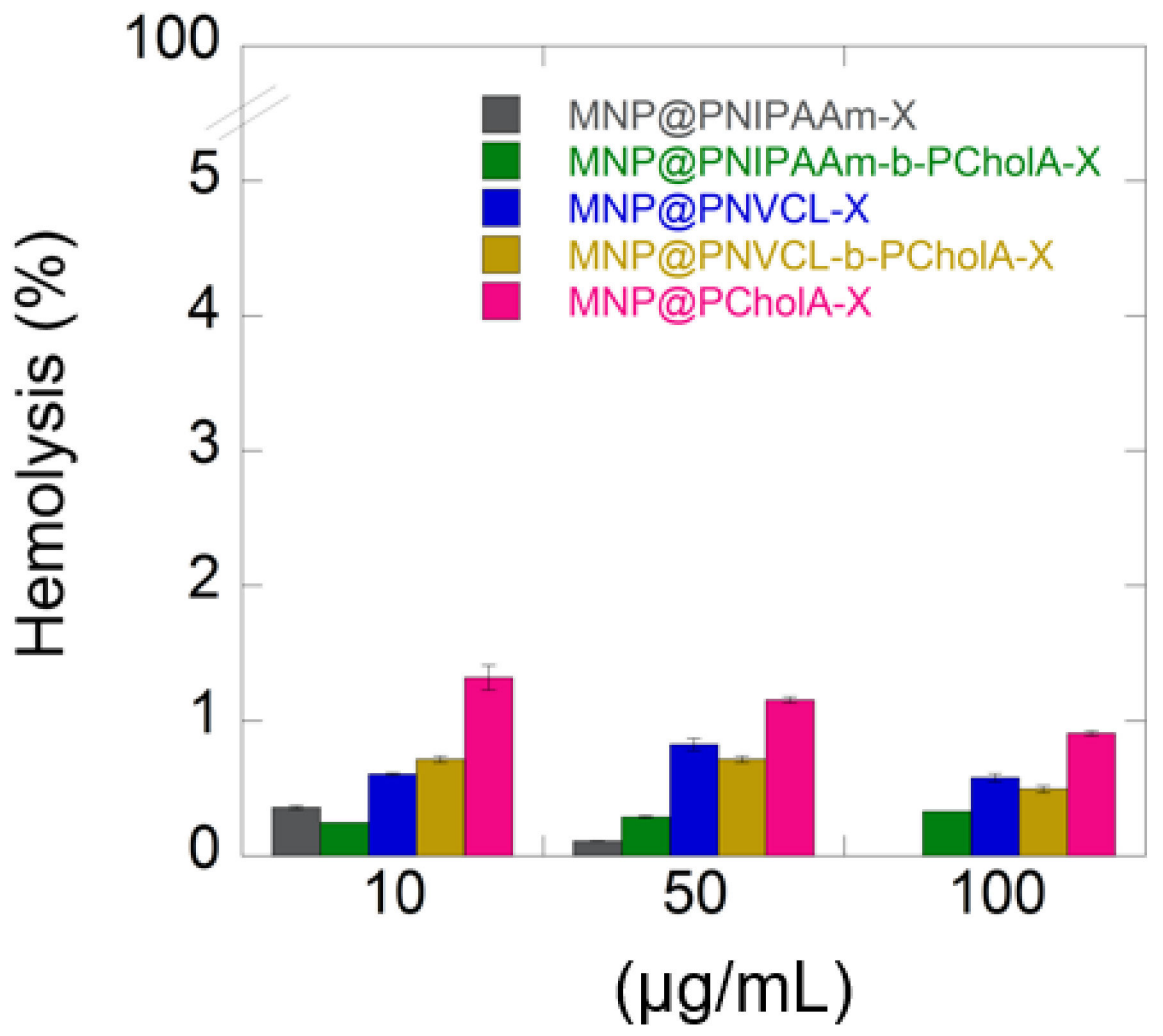
Hemocompatibility of polymer–iron oxide hybrids.

In this work, the hemocompatibility of synthesized particles was analyzed. The results presented in Figure 4 indicate that the tested polymer–iron oxide hybrids exhibit high compatibility with the exposed cells. For all the tested agents and concentrations, hemolysis below 5% was noted, indicating that the hybrids met the criteria for blood-contacting materials [24].

**Figure 5:**
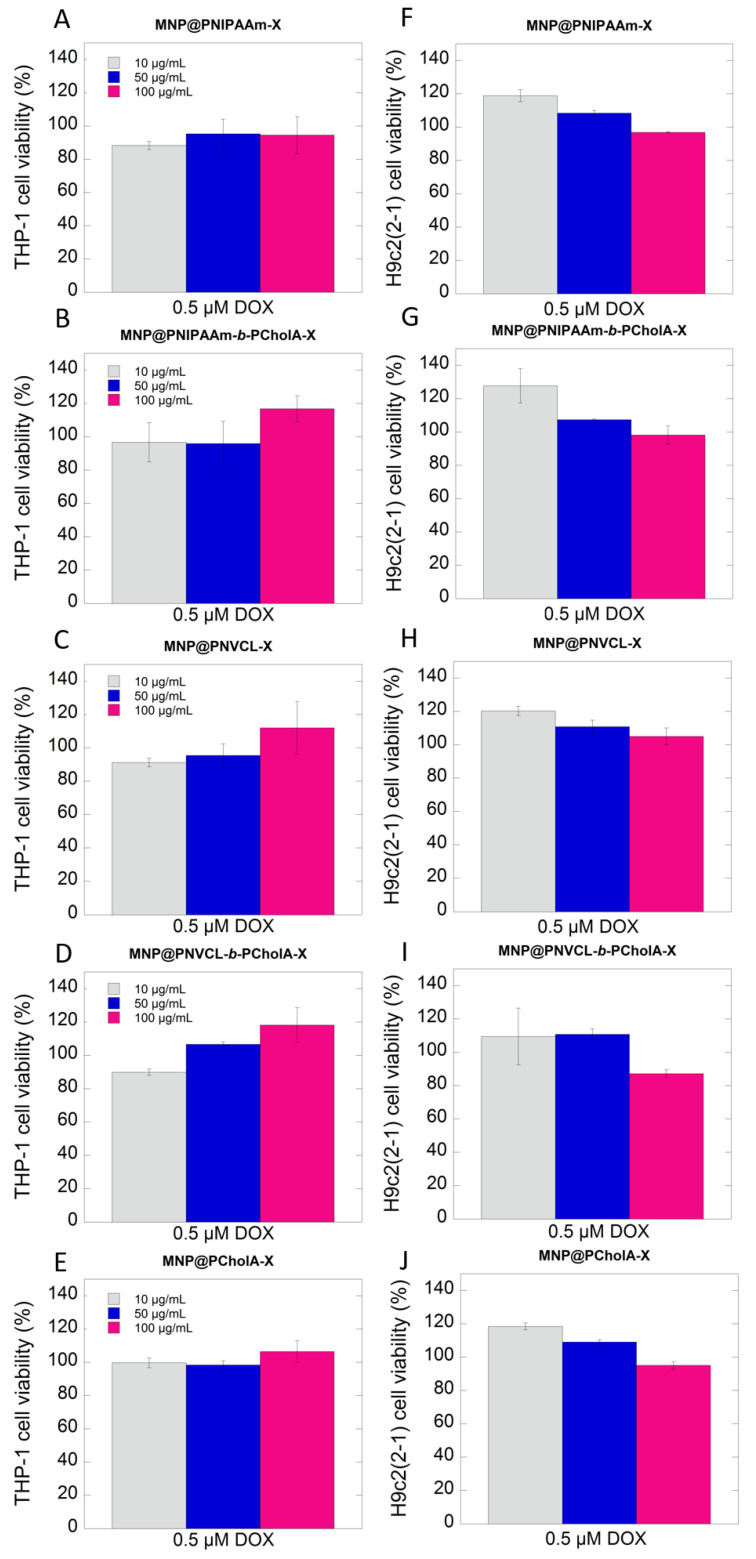
Lack of toxicity of the mixtures of DOX and polymer–iron oxide hybrids against representative immune THP-1 cells (**A**–**E**) and cardiomyocyte H9c2(2-1) cells (**F**–**J**).

The impact of the mixtures of doxorubicin (at constant concentration equal to 0.5 µM) and iron oxide-based hybrids (applied at the concentration range from 10 to 100 µg/mL) on the viability and metabolic activity of the human monocytic THP-1 cell line as well as on cardiomyocyte cells was tested. To explore the effect of the applied agents on representative immune cells, the colorimetric method based on the reduction of tetrazolium salt to formazan by living cells was employed. The obtained results indicated that the tested materials did not affect viability and metabolic activity of the treated cells. In all the analyzed cases, the viability of THP-1 cells was around 100% (Figure 5A–E). The effect of the mixtures on cardiomyocyte cells was studied by the neutral red assay. As shown in Figure 5F–J, the data indicate lack of toxic effect of the tested materials against the treated cells. Slight decrease of viability ~10% was indicated only after addition of a hybrid at the concentration of 100 µg/mL.

**Figure 6:**
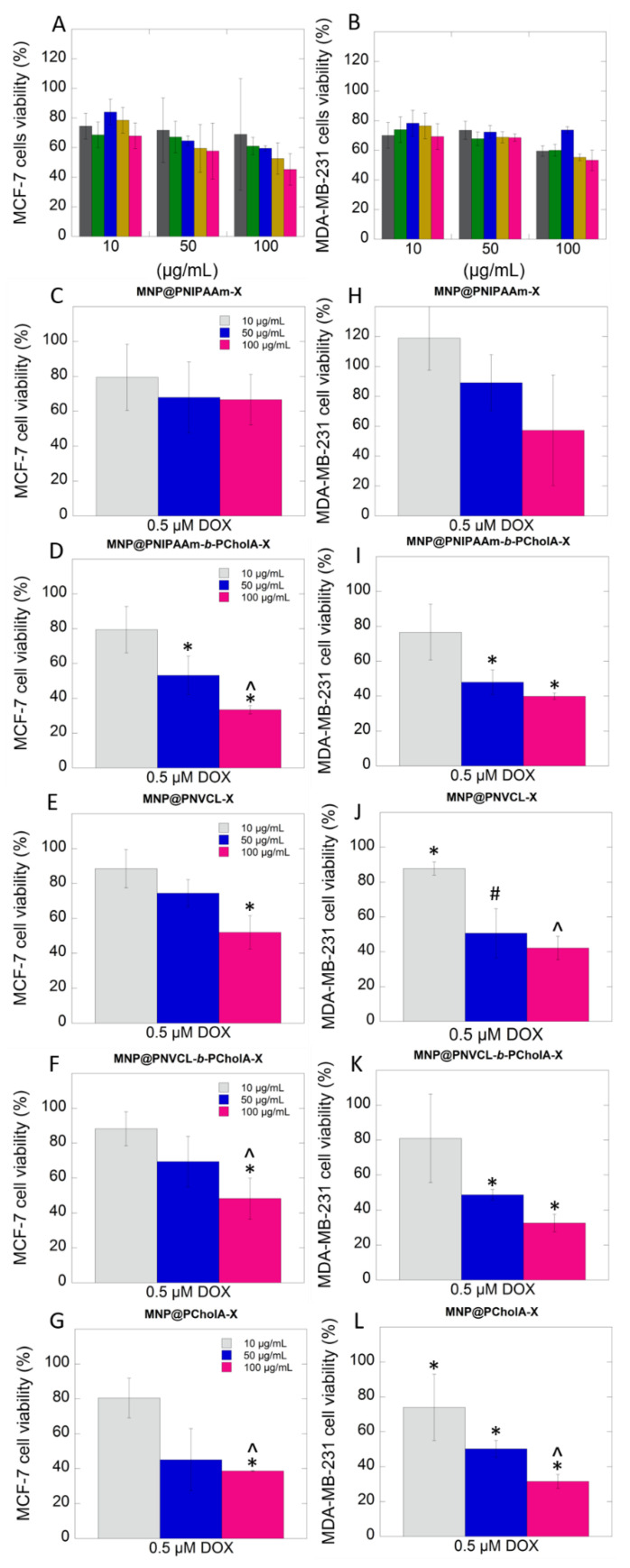
Toxic effect of the polymer–iron oxide hybrids against estrogen-dependent and estrogen-independent breast cancer cells. Viability of estrogen-dependent (**A**) and estrogen-independent (**B**) breast cancer cells in the presence of the polymer–iron oxide hybrids. Cytotoxic effect of the mixtures of DOX and polymer–iron oxide hybrids against estrogen-dependent (**C**–**G**) and estrogen-independent (**H**–**L**) breast cancer cells. Statistical significance for the tested hybrid vs. control was marked with (*); dose-dependent effect: 10 µg/mL vs. 50 µg/mL marked with (#) and 10 µg/mL vs. 100 µg/mL marked with (^), *p* ≤ 0.05. The data presented constitute average results from three measurements ± SD.

In the next step, the potential of magnetic particles coated by polymeric shells to sensitize breast cancer cells to doxorubicin was tested. Two different breast cancer cell lines, MCF-7 and MDA-MB-231, were incubated alone, with polymer-coated particles (applied at the concentration of 10, 50, and 100 µg/mL) or with a mixture of doxorubicin (at a constant concentration of 0.5 μM), and with polymer-coated particles (applied at the concentration of 10, 50 and 100 µg/mL) for 24 h. As shown in Figure 6A,B, magnetic hybrids comprising PNIPAAm or PNVCL did not induce a high toxic effect against both tested cell lines, cell viability around 80% was determined. However, the presence of poly(cholesteryl acrylate) block caused reduction in cell viability, even up to 60% at the concentration of 10 and 50 µg/mL and around 50% for the concentration of 100 µg/mL. Based on the results presented in Figure 6C,E, the mixtures of DOX with magnetic hybrids comprising PNIPAAm or PNVCL are characterized by relatively low toxicity against MCF-7 cells at the doses of 10 and 50 µg/mL. However, MNP@PNVCL-*b*-PCholA-X applied at the highest concentration (100 µg/mL) caused 50% depletion of cell viability. In the case of the MDA-MB-231 cell line, the dose-dependent effect was noted (Figure 6H,J). Viability of the cancer cells after treatment with the mixtures containing the highest dose of hybrids was established at 60% and 40% for MNP@PNIPAAm-X and MNP@PNVCL-X, respectively. The presence of cholesteryl moieties significantly affected the viability of both tested cell lines. As observed in Figure 6D,F, the viability of estrogen-dependent breast cancer cells incubated with the mixture of DOX and nanohybrids at the concentrations of 50 µg/mL and 100 µg/mL was significantly reduced to 55% and 35% (MNP@PNIPAAm-*b*-PCholA-X) and to 65% and 45% (MNP@PNVCL-*b*-PCholA-X). In the case of the MDA-MB-231 cell line, viability was reduced to 45% for MNP@PNIPAAm-*b*-PCholA-X and to 40% for MNP@PNVCL-*b*-PCholA-X. However, the best efficacy was observed after application of DOX with hybrids comprising only the poly(cholesteryl acrylate) shell. For both tested cell lines, incubation with the mixture of DOX and MNP@PCholA-X caused depletion of viability around 40%, even at the concentration of 50 µg/mL.

## 3. Discussion

Doxorubicin is commonly used in the treatment of several types of cancer including breast, gastric, lung, ovarian, thyroid, non-Hodgkin and Hodgkin lymphoma, multiple myeloma, sarcoma, and pediatric cancer [25]. However, administration of doxorubicin is associated with many adverse reactions which include fatigue, alopecia, nausea and vomiting, and oral sores. The major side effect of DOX is significant cardiac toxicity, which limits the long-term use of the drug [13]. Additionally, suppression of bone marrow, immune system disorders, and an increased risk of secondary malignancy development might occur.

It is established that red blood cells are crucial elements maintaining proper flow characteristics in microcirculation [26]. Different factors are noted as inductors of erythrocyte destruction such as presence of active bacterial infection, intrinsic defects of erythrocyte membranes, presence of exogenous materials, mechanical shear due to blood flow, pH and osmotic changes in blood [27,28]. Moreover, hemoglobin is released out as a consequence of intravenous and long-term administration of antineoplastic agents. In effect, the patients were diagnosed with hemolytic anemia, which is a generally uncommon complication during cancer treatment [29,30]. Thus, assessment of the erythrocyte lysis is a good indicator of the toxicity of any material external to the host cells. The results of hemocompatibility tests presented in Figure 4 indicate that tested polymer–iron oxide hybrids proposed as sensitizers of cancer cells exhibit high compatibility with the exposed cells meeting the criteria for blood-contacting materials [24]. Therefore, the tested materials might be used in combination therapy with doxorubicin, which is typically applied in the form of an intravenous injection.

In the second step of this study, the impact of the mixtures of doxorubicin (at a constant concentration equal to 0.5 µM) and iron oxide-based hybrids (applied at the concentration range from 10 to 100 µg/mL) on viability and metabolic activity of the human monocytic THP-1 cell line as well as on cardiomyocyte cells was tested. The aforementioned cells represent organs affected the most during DOX-based therapy. The obtained results indicated that the tested materials did not affect viability and metabolic activity of the treated cells. These results suggest that the combination of chemotherapeutic agents with nanosystems containing cholesterol moieties restrict its cytotoxic activity, and in effect might limit side effects of cancer treatment.

The published studies have shown that nanoparticles hold promise for a variety of biomedical applications including therapeutic and diagnostic levels [31,32]. Some reports also indicated that they might help to overcome resistance and sensitize cancer cells to classical chemotherapeutic agents [33,34]. Therefore, in the next step, we evaluated the potential of magnetic particles coated by polymeric shells containing poly(cholesteryl acrylate) to sensitize breast cancer cells to doxorubicin. For this purpose, two different breast cancer cell lines, MCF-7 and MDA-MB-231, were examined. Based on the obtained results, it can be concluded that the presence of cholesteryl moieties markedly enhanced biological activity of the hybrids, which resulted in stronger anti-cancer activity. To understand higher activity of DOX mixed with MNP@PCholA as compared to systems containing MNP@PNIPAAm-*b*-PCholA-X or MNP@PNVCL-*b*-PCholA-X, further investigations are needed. However, it can be supposed that this effect is related to several factors, such as zeta potential value, differences in hydrodynamic diameter, effects related to the presence/absence of the PNIPAAm/PNVCL layer, the number and availability of cholesteryl moieties present at the surface of the particles. It is in line with our previous studies, which showed that homopolymers bearing a cholesteryl moiety can disrupt plasma membranes and cause leakage of lactate dehydrogenase (LDH) from treated neoplastic cells [11]. It should be emphasized that, based on our results (Figure S6) and previously published studies, the viability of breast cancer cells after treatment with DOX at the concentration of 0.5 µM caused only a slight reduction of viability (10–15%) compared to untreated cells [35]. Furthermore, previously published results indicate that intratumor implantation of maghemite-based nanoparticles followed by exposure to a static magnetic field and doxorubicin caused reduction of cell viability to 60% [36]. Moreover, the anti-proliferative effect of this treatment procedure via induction of apoptotic cell death was revealed. Taking all this into account, the combination of chemotherapeutic agents with polymer–magnetic hybrids might provide a promising approach in the treatment of patients with breast cancer and for those refractory to doxorubicin. In the other study, the effect of PLGA nanocapsules loaded with MNPs and selol treatment on the viability of the MCF-7 cell lines was tested with or without subsequent exposure to the external alternating magnetic field (AMF). The results showed that the presence of AMF caused a 50% decrease in cell viability [37]. Wang et al. investigated the antitumor effect of a nanoparticle-based delivery system containing cross-linked hyaluronic acid and a low dose of DOX (X-NP-DOX) in both in vitro and in vivo conditions. It was indicated that the tested system has the ability to inhibit MCF-7 breast cancer cell growth, invasion, and migration. Moreover, it was also demonstrated that low doses of DOX when combined with a nanosystem inhibited Notch1 and Ras/MAPK pathways, decreased cancer stem cell population, and reduced tumorigenesis as compared to free DOX in both in vitro and in vivo settings [38]. In turn, Tomankova et al. performed a painstaking study to understand behavior of healthy and cancer cells after administration of superparamagnetic iron oxide (SPIO) colloidal nanoassemblies loaded with DOX compared with free DOX. The authors showed that different pathways to induce a cytotoxic effect on treated cells are engaged for the tested DOX nanosystem and the drug in free form. The explanation for the differences observed in cytotoxic effects of DOX and SPIO–DOX systems may be different mechanisms of their transport as well as intracellular localization. This study strongly support our findings indicating that application of DOX in combination with iron oxide particles with cholesterol moieties could reduce the risk of free DOX toxicity [35].

## 4. Materials and Methods

### 4.1. Materials

Iron (III) chloride hexahydrate FeCl_3_·6H_2_O and iron (II) chloride tetrahydrate FeCl_2_·6H_2_O (Sigma-Aldrich, St. Louis, MO, USA), 3-aminoproplotrimethoxysilane (APTMS, 97%, Sigma-Aldrich, St. Louis, MO, USA), 2-bromopropionyl bromide (97%, Sigma-Aldrich, St. Louis, MO, USA), carbon disulfide (CS_2_, ≥ 99.9%, Sigma-Aldrich, St. Louis, MO, USA), potassium hydroxide (KOH, pure, Avantor Performance Materials Poland S.A., Gliwice, Poland), triethylamine (Et_3_N, Avantor Performance Materials Poland S.A., Gliwice, Poland), acryloyl chloride (96%, Alfa Aesar, Ward Hill, MA, USA) were used as received. The initiator, 2,2′-azobis(2-methylpropionitrile) (AIBN, ≥ 98%, Sigma-Aldrich, St. Louis, MO, USA), was recrystallized from methanol. *N*-vinylcaprolactam (NVCL, > 98%, TCI, Tokyo, Japan) was recrystallized from hexane prior to use and stored in a freezer. *N*-isopropylacrylamide (NIPAAm, 99 %, Acros Organics, Geel, Belgium) was recrystallized from toluene–hexane (60:40, *v/v*) prior to use. Potassium *O*-ethyl carbonodithioate (KSCSOEt) was synthesized according to the well-known procedure [11]. All organic solvents were purchased from Avantor Performance Materials Poland S.A. (Gliwice, Poland) and were distilled before use.

### 4.2. Methods

#### 4.2.1. Physicochemical Characterization

The ^1^H and 13C NMR spectra were recorded on Bruker Avance II 400 or Avance DPX 200 spectrometers (Billerica, MA, USA) operating at 400 and 100 MHz, respectively, using CDCl_3_ solutions. ATR–FTIR spectra were recorded using a Nicolet 6700 FTIR Spectrometer from Thermo Fisher Scientific (Waltham, MA, USA) equipped with an ATR accessory. The spectra were collected in the wavenumber range of 4000 to 500 cm^−1^ by coadding 32 scans with a resolution of 4 cm^−1^. Thermogravimetric analyses (TGA) were performed on a Mettler Toledo Star TGA/DSC unit (Greifensee, Switzerland). Samples weighing 2–3 mg were placed in aluminum oxide crucibles and heated from 50 °C to 900 °C at a heating rate of 10 K min^−1^ under an argon flow rate of 40 mL·min^−1^. The formation of magnetic particles, particle size and morphology were confirmed by transmission electron microscopy (TEM) (Tecnai G2 X-TWIN, FEI Company, Hillsboro, OR, USA). Samples for TEM were prepared on holey carbon copper grids. Dynamic light scattering measurements were performed using a Zetasizer Nano-ZS (Malvern Instruments, UK) equipped with a 4 mW helium/neon laser (l = 633 nm) and a thermoelectric temperature controller. All the measurements were performed at 25 °C with a backscatter detection system at 173°. The samples were dispersed in water to reach a final concentration of 0.25 mg·mL^−1^. The particle sizes are expressed as the mean hydrodynamic diameter of five measurements. Magnetic properties of the nanoparticles MNP@NH2, MNP@PNIPAAm and MNP@PNIPAAm-b-PcholA were studied using a Quantum Design MPMS 5XL SQUID-type magnetometer in the temperature range 5–400 K and in magnetic fields up to 10 kOe. Each sample was placed in a standard gelatin capsule and immobilized with Varnish glue for the measurement. All the data were carefully corrected for the diamagnetic contribution of the holder.

#### 4.2.2. Biological Studies

Cell culture: The non-adherent human monocytic cell line THP-1 (ATCC, TIB-202) was cultured in the Roswell Park Memorial Institute medium (RPMI-1640, ATCC) supplemented with 10% heat-inactivated fetal bovine serum (EURx, Gdansk, Poland), 1% penicillin/streptomycin mixture (Gibco, Life Technologies, Germany), and 2-mercaptoethanol to a final concentration of 0.05 mM (Gibco, Life Technologies, Germany). Estrogen-dependent MCF-7, estrogen-independent MDA-MB-231 human breast cancer cells and rat myocardial H9c2(2-1) cells were obtained from ATCC and maintained in the Dulbecco’s modified Eagle’s medium (DMEM) with 10% heat-inactivated fetal bovine serum and 100 μg/mL penicillin/streptomycin at 37 °C in a humified atmosphere containing 5% CO_2_. The cells were kept in culture until passage 10 and analyzed from passages 8–10. For experiments, the cells were plated in 96-well plates at a seeding density of 104/0.32cm^2^ and cultured to reach confluency of 80%.

Hemocompatibility assessment: Hemocompatibility was examined using fresh human red blood cells (RBCs) suspended in the phosphate-buffered saline (PBS) (hematocrit ~5%). The tested carriers were added at the concentration range from 10 to 100 μg/mL and incubated for 1 h at 37 °C. In the next step, the relative hemoglobin concentration in supernatants after centrifugation at 2500 g was spectrophotometrically measured at wavelength 540 nm. One hundred percent hemolysis was taken from samples in which 1% Triton X-100 was added to disrupt all cell membranes, while 0% hemolysis was taken from samples after the addition of 10 μL PBS.

Cell viability and metabolic activity: The viability of MCF-7 and MDA-MB-231 breast cancer cells as well as H9c2(2-1) cardiomyocyte cells were measured using a Neutral Red assay. In brief, polymer-coated magnetic particles were added to cells at the concentrations of 10, 50, or 100 µg/mL, while doxorubicin was added at the concentration of 0.5 µM and incubated. After 24 h exposure, the determination of toxic endpoints was carried out by spectrophotometric methods. For this purpose, the neutral red solution (0.33%) was added to each well. The mixture was incubated for 2 h and neutral red was removed. In the next step, the cells were quickly rinsed with a Neutral Red assay fixative. Then, the fixative solution was removed, and the incorporated dye was then solubilized in a volume of the solubilization solution. The absorbance at a wavelength of 540 nm was measured and calculated as a percentage of control.

In the other experiment, viability and metabolic activity of monocytic THP-1 cells was determined by the MTS assay. After 24 h exposure, 20 µL MTS reagent was added to each well. Then, the cells were incubated for 2 h in the dark at 37 °C with a 5% CO_2_ atmosphere. The formazan absorbance was measured at 490 nm using a Biotek microplates reader. The mean absorbance of the untreated cells served as the reference for calculating 100% cellular viability. To determine viability, the results were calculated as a percentage of viability of the untreated cells.

In all biological experiments, the obtained data were corrected to exclude the effect of optical interference related to absorbance of iron oxide-based nanoparticles.

Statistical analysis: Statistical analyses were performed using the Statistica 13.3 software (StatSoft Inc., Tulsa, OK, USA). The data were analyzed using standard statistical analyses, including the Student’s’ *t*-test (for independent samples). *p*-values lower than 0.05 were considered significant.

## 5. Conclusions

The presented results indicate that doxorubicin-based chemotherapy may be improved by combination with polymer–iron oxide hybrids. The performed studies demonstrated that nanoparticle-based systems containing cholesteryl moieties significantly improve the killing efficiency of low-dose doxorubicin, decreasing the viability of cancer cells even up to 40%. It is postulated that the presence of cholesteryl moieties due to the high affinity of this molecule to cell membranes sensitizes cancer cells to doxorubicin and enhances the drug uptake. Additionally, high compatibility and lack of toxicity of the mixtures of DOX and magnetic hybrids against human red blood cells, monocytic THP-1 cells, and cardiomyocyte H9C2(2-1) cells, which are the most exposed during standard chemotherapy, were observed. Due to improved efficacy, high targetability to neoplastic cells, and compatibility with host cells, application of a low-dose DOX with polymer–iron oxide hybrids, which are relatively easy to synthetize and safe when intravenously administered, might be a promising approach for breast cancer treatment.

## Figures and Tables

**Table 1:** Polymeric shells formed on the surface of MNP@X.

Nanohybrids	Type of Monomer		TG Weight Loss at 1120 K
	R1	R2	(%)
MNP@PNIPAAm-X	** 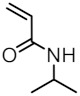 **	-	25
MNP@PNIPAAm-*b*-PCholA-X	** 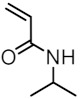 **	** 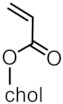 **	34
MNP@PNVCL-X	** 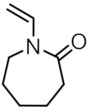 **	-	19
MNP@PNVCL-*b*-PCholA-X	** 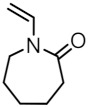 **	** 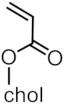 **	25
MNP@PCholA-X	** 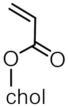 **	-	16

## Data Availability

The data are contained within the supplementary material or are available from the corresponding authors.

## Supplementary Materials

The following are available online at https://www.mdpi.com/article/10.3390/ijms22094898/s1: synthetic procedures; Figure S1. ^1^H NMR spectrum of cholesteryl acrylate (CholA); Figure S2. Zero-field-cooled/field-cooled magnetization for modified iron oxide nanoparticles; Figure S3. Hysteretic loops for modified iron oxide nanoparticles at varying temperatures; Figure S4. Magnetization of samples at 300 K with fits of law of approach to saturation; Table S1. The summary of the data calculated based on magnetization measurements; Figure S5. Representative DLS data obtained for polymer-modified particles; Figure S6. Toxic effect of doxorubicin applied at the concentration of 0.5µM against tested cell lines.
