# Supplementary material for: Magnetic Particles with Polymeric Shells Bearing Cholesterol Moieties Sensitize Breast Cancer Cells to Low Doses of Doxorubicin
Source: Int J Mol Sci. 2021 May 5;22(9):4898. doi: 10.3390/ijms22094898 (PMC8125246; doi:10.3390/ijms22094898)
Supplement: Supplementary file 1 [file ijms-22-04898-s001.zip › ijms-1155983-supplementary.pdf]

Supplementary Materials

Magnetic particles with polymeric shells bearing cholesterol moieties sensitize breast cancer cells to low doses of doxorubicin

Karolina H. Markiewicz[1*], Katarzyna Niemirowicz-Laskowska[2*], Dawid Szymczuk[1,3], Kacper Makarewicz[1], Iwona Misztalewska-Turkowicz[1], Przemysław Wielgat[4], Anna M. Majcher-Fitas[5], Sylwia Milewska[2], Halina Car[2], Agnieszka Z. Wilczewska[1*]

1. Faculty of Chemistry, University of Bialystok, Ciolkowskiego 1k, 15-245 Bialystok, Poland, k.markiewicz@uwb.edu.pl, agawilcz@uwb.edu.pl.
2. Department of Experimental Pharmacology, Medical University of Bialystok, Szpitalna 37, 15-361 Bialystok, Poland, katarzyna.niemirowicz@umb.edu.pl.
3. Doctoral School of Exact and Natural Sciences, University of Bialystok, Bialystok, Poland.
4. Department of Clinical Pharmacology, Medical University of Bialystok, Waszyngtona 15A, 15-274 Bialystok, Poland.
5. Faculty of Physics, Astronomy and Applied Computer Science, Jagiellonian University, Łojasiewicza 11, 30-348 Krakow, Poland.

Synthetic procedures

Cholesteryl acrylate: Cholesterol (5 g, 12.9 mmol), 350 mL of anhydrous dichloromethane, and triethylamine (2.51 mL, 18.1 mmol) were placed in a flask. The mixture was placed on a magnetic stirrer in an ice bath (0 °C), under an inertflow of argon, and protected from light. Then acryloyl chloride (1.28 mL, 15.5 mmol) was added dropwise. After 24h of stirring, the mixture was washed three times with 5% hydrochloric acid. The product was precipitated in ethanol and filtered. 3 g of white powder was obtained. **CholA** ^{1}H NMR δ_H (400 MHz, $CDCl_3$): 6.40 (d, J = 17.3 Hz, 1H), 6.11 (dd, J = 17.3, 10.4 Hz, 1H), 5.81 (d, J = 10.4 Hz, 1H), 5.40 (d, J = 4.4 Hz, 1H), 4.70 (m, 1H), 2.37 (d, J = 7.7 Hz, 2H), 1.04 (s, 3H), 0.93 (d, J = 6.5 Hz, 3H), 0.87 (dd, J = 6.6, 1.4 Hz, 6H), 0.69 (s, 3H) (Figure S1).

MNP: $FeCl_2{\cdot}4H_2O$ (2.15 g, 10.8 mmol) and $FeCl_3{\cdot}6H_2O$ (5.8 g, 21.6 mmol) were placed in two flasks, dissolved in 200 mL of deionized water each, and degassed by bubbling argon for 30 min. $FeCl_3$ and $FeCl_2$ solutions were mixed at room temperature, and placed in an ultrasound bath at 80 °C. The ammonia solution (25%) was added dropwise to reach pH = 9. After 30 min of sonicating at 80 °C, the mixture was cooled down. The product was separated by magnetic decantation and washed several times with deionized water. The particles were redispersed in 125 mL of ethanol and stored in suspension (~17 mg·mL^{-1}). **MNP** FT IR (ν_{max}, cm^{-1}): 3434 (O-H), 538 (Fe-O).

MNP@NH$_2$: 125 mL of MNP@OH suspension in ethanol (around 2 g of MNP@OH) was redispersed in 1.5L of ethanol, sonicated for 1 h, and degassed by bubbling argon for 30 min. The ammonia solution (25%) was added dropwise to reach pH = 9, then, APTMS (1.5 mL, 8.6 mmol) was added. The mixture was mechanically stirred for 4 h. The product was separated by magnetic decantation and washed several times with ethanol. The particles were stored in

ethanol. **MNP@NH$_2$** FT IR (ν_{max}, cm^{-1}): 3500-3200 (N-H), 2925 (C-H), 1560 (N-H$_{def}$), 1004 (Si-O-Si), 547 (Fe-O).

MNP@Br: 125 mL of MNP@NH$_2$ suspension in ethanol (around 2 g of MNP@NH$_2$) was redispersed in dichloromethane in an ultrasound bath. The particles were separated from the solvent by magnetic decantation, the cycle was repeated several times. Then, the particles were redispersed in anhydrous dichloromethane (800 mL), sonicated and degassed by bubbling argon for 30 minutes. Triethylamine (10.4 mL, 75.2 mmol) was added, and next 2-bromopropionyl bromide (8 mL, 75.2 mmol) diluted in 20 mL of dried DCM was added dropwise. The mixture was sonicated for 15 min under argon atmosphere. The product MNP@Br was separated by magnetic decantation and washed several times with DCM. The obtained particles were redispersed in 125 mL of ethanol and stored in suspension. **MNP@Br** FT IR (ν_{max}, cm^{-1}): 3500-3200 (N-H), 2934 (C-H), 1735 (C=O), 1654 (N-H) 1034 (Si-O-Si), 549 (Fe-O).

MNP@X: 125 mL of MNP@Br suspension in ethanol (2 g) was diluted in 1,5L of ethanol, sonicated in an ultrasound bath and degassed by bubbling argon for 30 min. Then, ethyl potassium dithiocarbonate (2 g, 12.4 mmol) was added in portions and the mixture was mechanically stirred for 18 hour. The product was separated by magnetic decantation and washed several times with ethanol. The particles were dispersed in 125 mL of ethanol and stored in suspension. **MNP@X** FT IR (ν_{max}, cm^{-1}): 3500-3200 (N-H), 2930 (C-H), 1646 (N-H), 1115 (C=S), 1041 (Si-O-Si), 546 (Fe-O).

MNP@PNIPAAm: 3.125 mL of MNP@X suspension in ethanol was magnetically separated. The particles were washed several times in THF. MNP@X (~50 mg), *N*-isopropylacrylamide (500 mg, 4.4 mmol), *O*-ethyl-*S*-(1-methoxycarbonyl)ethyldithio- carbonate (0.026 mg, 0.17 mmol) and dried tetrahydrofuran (1 mL) were placed in a flask, sonicated and degassed by bubbling argon for 30 min. Then, AIBN (4 mg, 0.024 mmol) was added (in two portions, at the beginning and after 2h of the reaction), and the solution was heated at 70°C for 4h. The polymerization was stopped by diluting the mixture in solvent (THF) and magnetic decantation. The cycle of redispersion and magnetic separation was repeated several times. The particles were dispersed in 50 mL of THF and stored in suspension (~1.4 mg·mL^{-1}). **MNP@PNIPAM** FT IR (ν_{max}, cm^{-1}): 3500-3200 (N-H), 2926 (C-H), 1638 (C=O), 1547 (N-H), 1036 (Si-O-Si), 546 (Fe-O).

MNP@PNIPAAm-*b*-PCholA: 35.7 mL of MNP@PNIPAAm suspension in ethanol was magnetically separated. The particles were washed several times in THF. MNP@PNIPAAm (~50 mg), cholesteryl acrylate (200 mg, 0.45 mmol), *O*-ethyl-*S*-(1-methoxycarbonyl)ethyldithiocarbonate (0.026 mg, 0.17 mmol) and dried tetrahydrofuran (1 mL) were placed in a flask, sonicated and degassed by bubbling argon for 30 min. Then, AIBN (4 mg, 0.024 mmol) was added (in two portions, at the beginning and after 12h of the reaction). The mixture was heated at 70°C for 24h, then the mixture was diluted in solvent (THF) and magnetically separated. The cycle of redispersion and magnetic separation was repeated several times. **MNP@PNIPAAm-*b*-PCholA** FT IR (ν_{max}, cm^{-1}): 3500-3200 (N-H), 2920 (C-H), 1704 (C=O), 1637 (C=O), 1546 (N-H), 1035 (Si-O-Si), 553 (Fe-O).

MNP@PCholA: 3.125 mL of MNP@X suspension in ethanol (~50 mg of MNP@X) was magnetically separated. The particles were washed several times in THF. MNP@X nanoparticles (~50 mg), cholesteryl acrylate (200 mg, 0.45 mmol), *O*-ethyl-*S*-(1-

methoxycarbonyl)ethyldithiocarbonate (0.026 mg, 0.17 mmol) and dried tetrahydrofuran (1 mL) were placed in a flask, sonicated and degassed by bubbling argon for 30 min. Then, AIBN (4 mg, 0.024 mmol) was added (in two portions, at the beginning and after 12h of the reaction), and the solution was heated at 70°C for 24h. The polymerization was stopped by diluting the mixture in solvent (THF) and magnetic decantation. The cycle of redispersion and magnetic separation was repeated several times. **MNP@PCholA** FT IR (ν_{max}, cm^{-1}): 3500-3200 (N-H), 2934 (C-H), 1651 (C=O), 1547 (N-H), 1125 (C-O-C), 551 (Fe-O).

MNP@PNVCL: 3.125 mL of MNP@X suspension in ethanol was magnetically separated. The particles were washed several times in THF. MNP@X nanoparticles (~50 mg), *N*-vinylcaprolactam (500 mg, 4.4 mmol), *O*-ethyl-*S*-(1-methoxycarbonyl)ethyldithiocarbonate (0.026 mg, 0.17 mmol) and dried tetrahydrofuran (1 mL) were placed in a flask, sonicated for 30 min, and degassed by bubbling argon for 30 min. Then, AIBN (4 mg, 0.024 mmol) was added (in two portions, at the beginning and after 2h of the reaction), and the solution was heated at 70°C for 4h. The polymerization was stopped by diluting the mixture in solvent (THF) and magnetic decantation. The cycle of redispersion and magnetic separation was repeated several times. The particles were dispersed in 50 mL of THF and stored in suspension (~1.19 $mg{\cdot}mL^{-1}$). **MNP@PNVCL** FT IR (ν_{max}, cm^{-1}): 3500-3200 (N-H), 2931 (C-H), 1642 (C=O), 1192 (C-N), 1036 (Si-O-Si), 549 (Fe-O).

MNP@PNVCL-*b*-PCholA: 42 mL of MNP@PNVCL suspension in ethanol (~50 mg of MNP@PNIPAAm) was magnetically separated. The particles were washed several times in THF. MNP@PNVCL (~50 mg), cholesteryl acrylate (200 mg, 0.45 mmol), *O*-ethyl-*S*-(1-methoxycarbonyl)ethyldithiocarbonate (0.026 mg, 0.17 mmol) and dried tetrahydrofuran (1 mL) were placed in a flask, sonicated for 30 min, and degassed by bubbling argon for 30 min. Then, AIBN (4 mg, 0.024 mmol) was added (in two portions, at the beginning and after 12h of the reaction), and the solution was heated at 70°C for 24h. The polymerization was stopped by diluting the mixture in solvent (THF) and magnetic decantation. The cycle of redispersion and magnetic separation was repeated several times. **MNP@PNVCL-*b*-PCholA** FT IR (ν_{max}, cm^{-1}): 3500-3200 (N-H), 2922 (C-H), 2852 (C-H), 1701 (C=O), 1641 (C=O), 1545 (N-H), 1117 (C-N), 1035 (Si-O-Si), 549 (Fe-O).

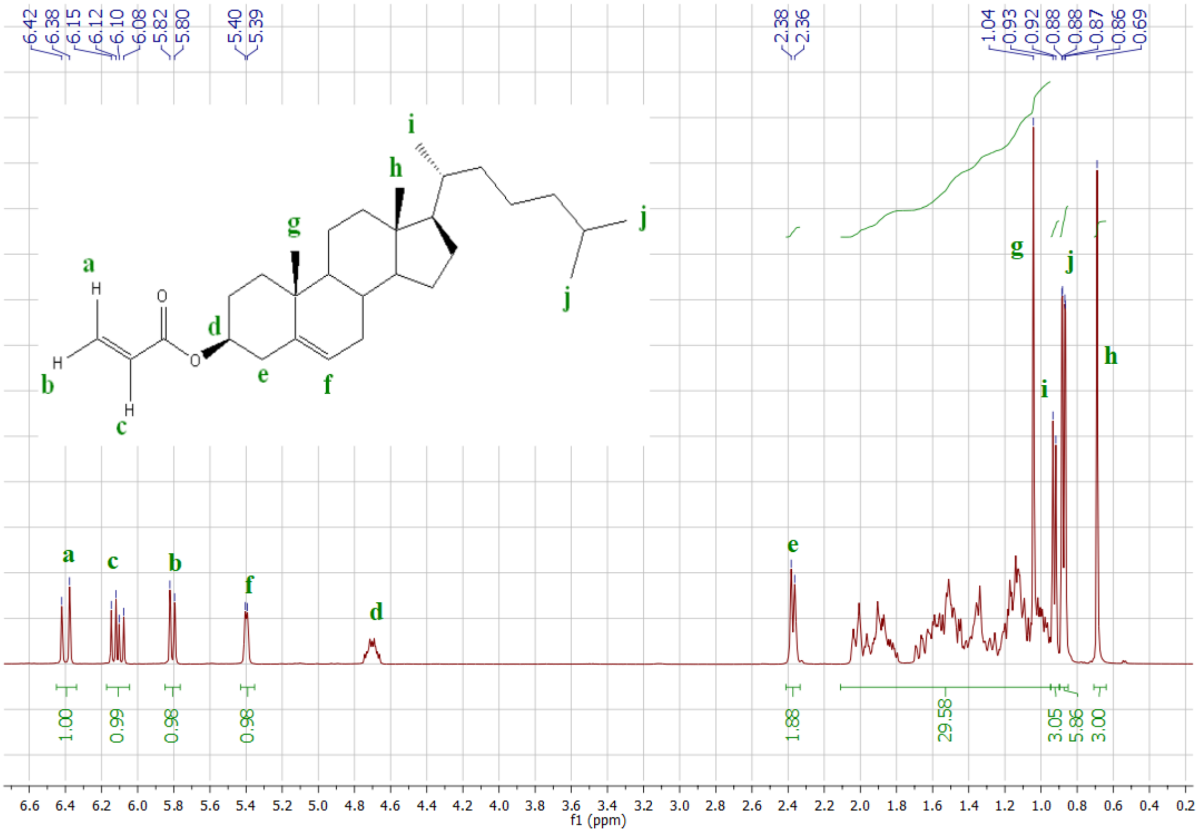

Figure S1. ^{1}H NMR spectrum of cholesteryl acrylate (CholA).

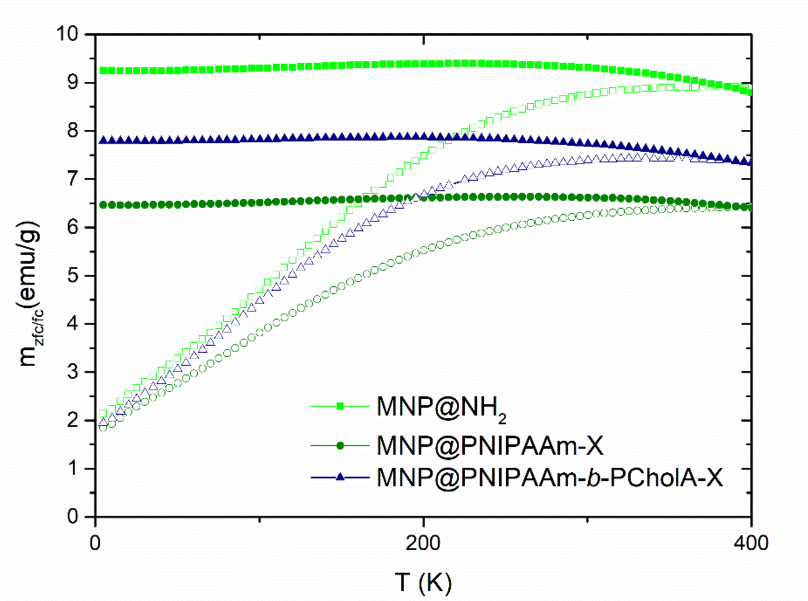

Figure S2. Zero field-cooled (hollow points))/field cooled (full points) magnetization for modified iron oxide nanoparticles. Measurements were done in 100 Oe.

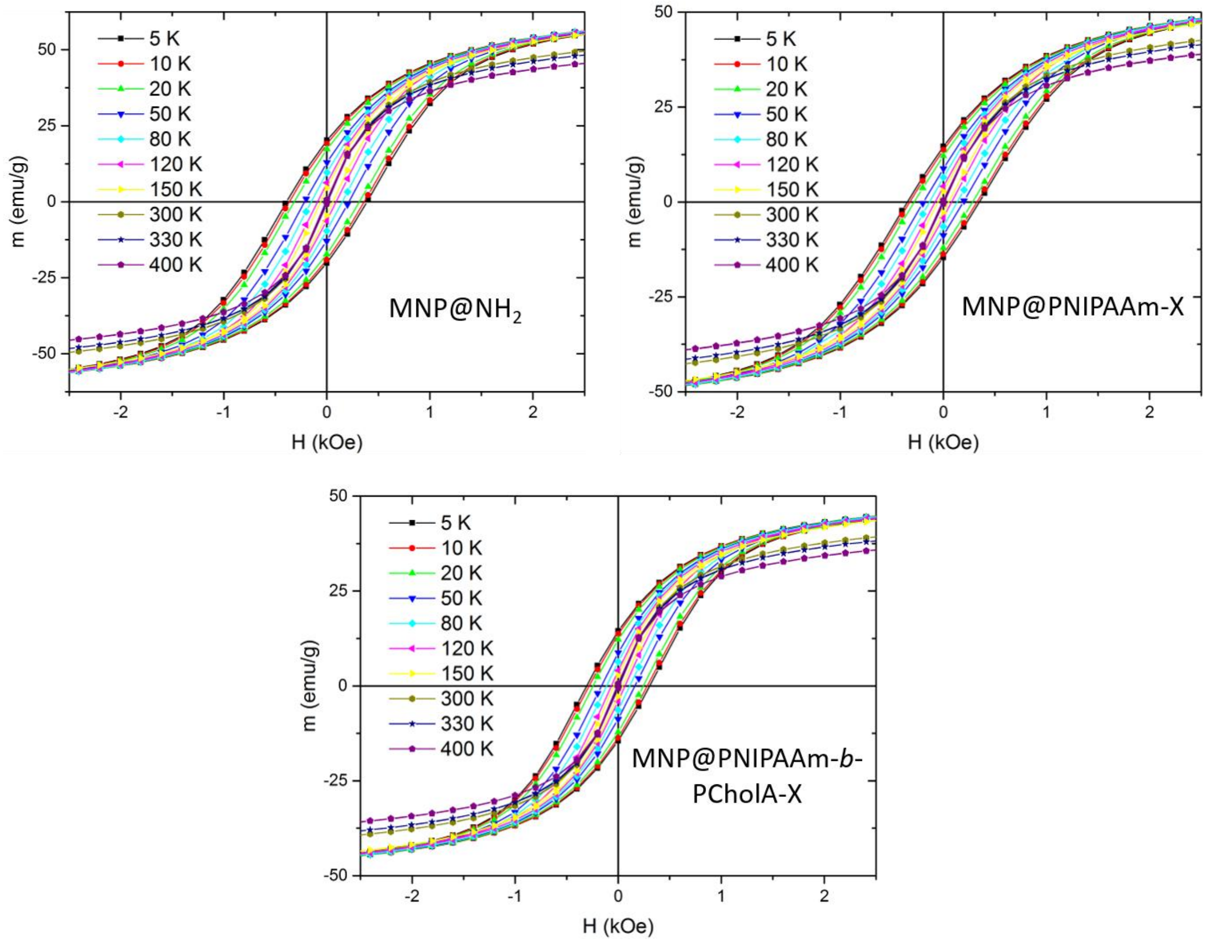

Figure S3. Hysteretic loops for modified iron oxide nanoparticles at varying temperatures.

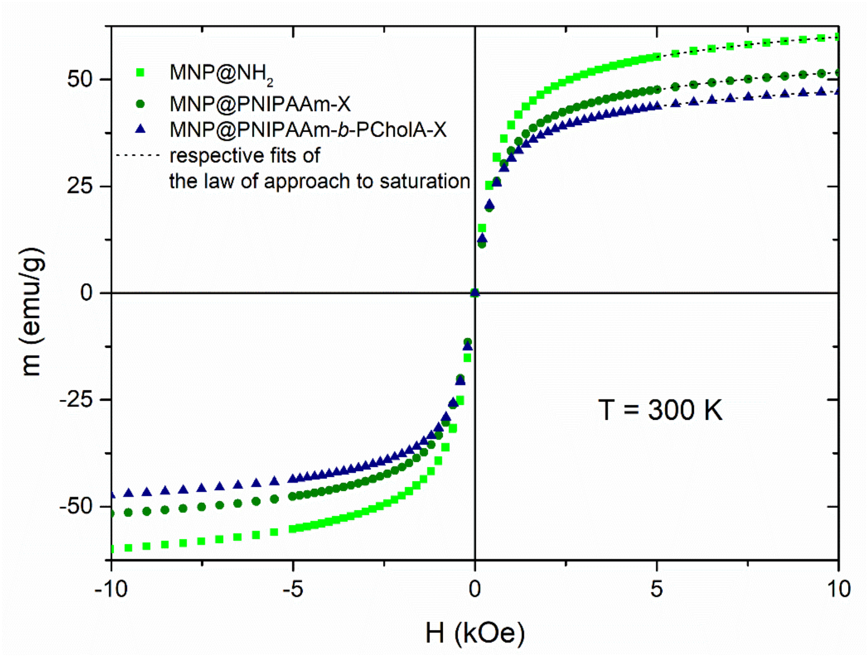

Figure S4. Magnetization of samples at 300 K with fits of law of approach to saturation.

Table S1. The summary of the data calculated based on the magnetization measurements.

Sample	μ/μ_B	D (nm)	M_S at 400 K (emu/g)	M_S at 300 K (emu/g)
**MNP@NH$_2$**	18480(660)	18(2)	60.5(1)	66.7(1)
**MNP@PNIPAAm -X**	17180(520)	17(2)	52.0(1)	57.5(1)
**MNP@PNIPAAm-*b*-PcholA-X**	19640(700)	17(2)	47.5(1)	52.7(1)

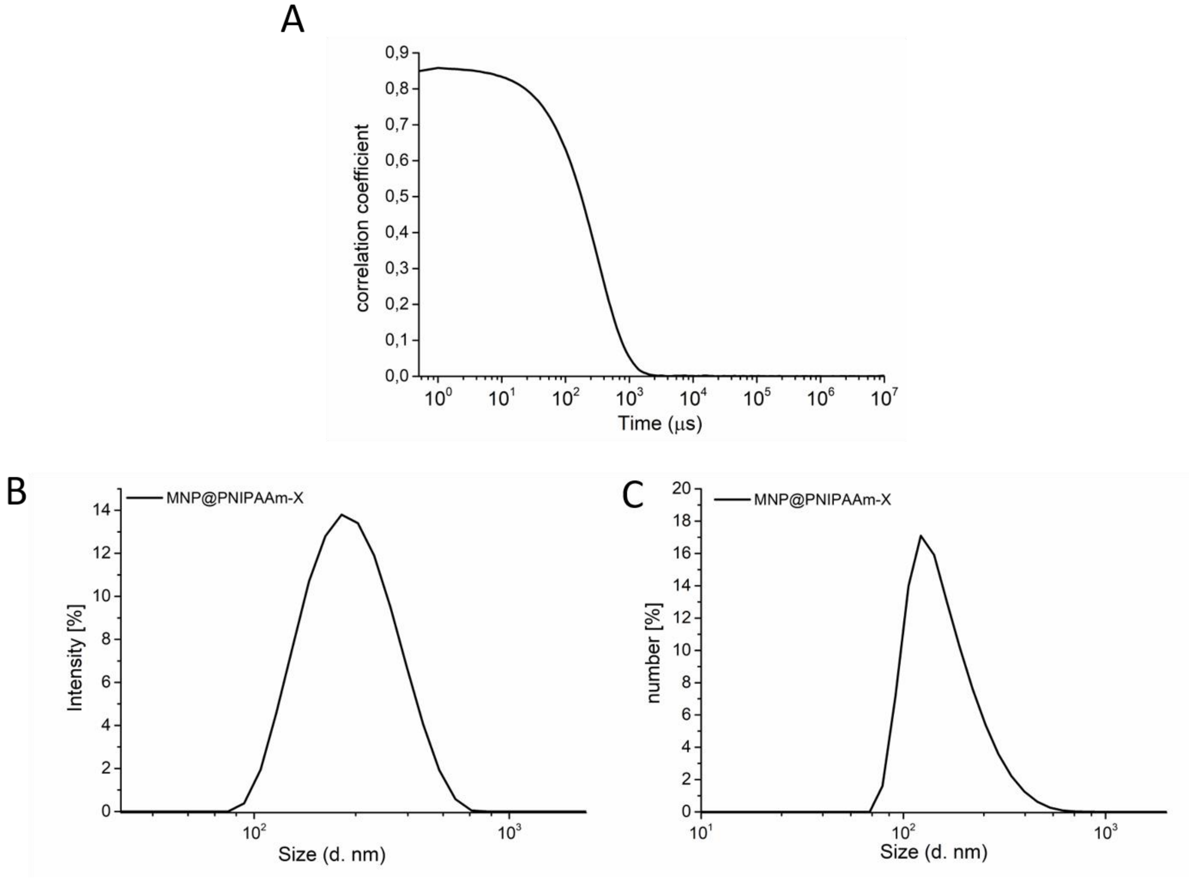

Figure S5. Representative DLS data obtained for polymer-modified particles.

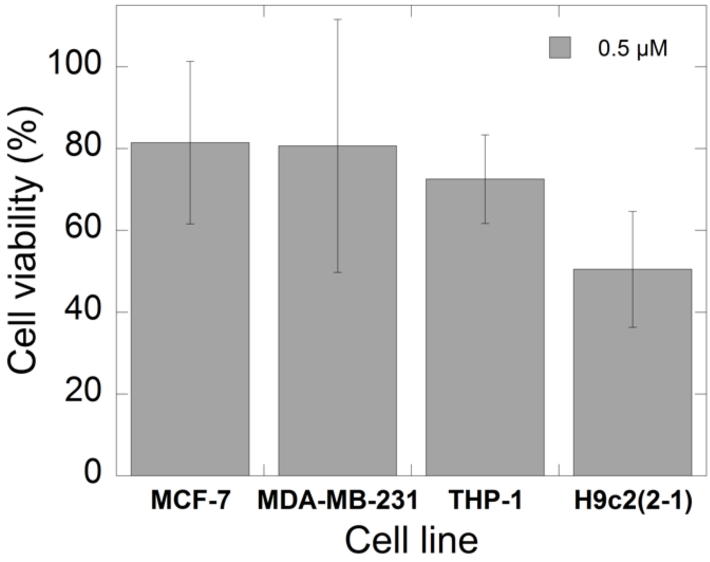

Figure S6. Toxic effect of doxorubicin applied at the concentration of 0.5µM against tested cell lines.
